# Latin America and the Caribbean SARS-CoV-2 Surveillance: Longitudinal Trend Analysis

**DOI:** 10.2196/25728

**Published:** 2021-04-27

**Authors:** Lori Post, Ramael O Ohiomoba, Ashley Maras, Sean J Watts, Charles B Moss, Robert Leo Murphy, Michael G Ison, Chad J Achenbach, Danielle Resnick, Lauren Nadya Singh, Janine White, Azraa S Chaudhury, Michael J Boctor, Sarah B Welch, James Francis Oehmke

**Affiliations:** 1 Buehler Center for Health Policy and Economics, Feinberg School of Medicine Northwestern University Chicago, IL United States; 2 Feinberg School of Medicine Northwestern University Chicago, IL United States; 3 Institute of Food and Agricultural Sciences University of Florida Gainsville, FL United States; 4 Institute of Global Health, Feinberg School of Medicine Northwestern University Chicago, IL United States; 5 Divison of Infectious Disease, Feinberg School of Medicine Northwestern University Chicago, IL United States; 6 International Food Policy Research Institute Washington DC, DC United States

**Keywords:** 7-day persistence, acceleration, Arellano–Bond estimator, COVID-19 surveillance system, COVID-19, dynamic panel data, econometrics, economic, generalized method of moments, global COVID-19 surveillance, Latin America and the Caribbean, longitudinal, metric, persistence, policy, public health surveillance, SARS-CoV-2, second wave, surveillance metrics, transmission deceleration, transmission jerk, transmission speed, trend analysis

## Abstract

**Background:**

The COVID-19 pandemic has placed unprecedented stress on economies, food systems, and health care resources in Latin America and the Caribbean (LAC). Existing surveillance provides a proxy of the COVID-19 caseload and mortalities; however, these measures make it difficult to identify the dynamics of the pandemic and places where outbreaks are likely to occur. Moreover, existing surveillance techniques have failed to measure the dynamics of the pandemic.

**Objective:**

This study aimed to provide additional surveillance metrics for COVID-19 transmission to track changes in the speed, acceleration, jerk, and persistence in the transmission of the pandemic more accurately than existing metrics.

**Methods:**

Through a longitudinal trend analysis, we extracted COVID-19 data over 45 days from public health registries. We used an empirical difference equation to monitor the daily number of cases in the LAC as a function of the prior number of cases, the level of testing, and weekly shift variables based on a dynamic panel model that was estimated using the generalized method of moments approach by implementing the Arellano–Bond estimator in R. COVID-19 transmission rates were tracked for the LAC between September 30 and October 6, 2020, and between October 7 and 13, 2020.

**Results:**

The LAC saw a reduction in the speed, acceleration, and jerk for the week of October 13, 2020, compared to the week of October 6, 2020, accompanied by reductions in new cases and the 7-day moving average. For the week of October 6, 2020, Belize reported the highest acceleration and jerk, at 1.7 and 1.8, respectively, which is particularly concerning, given its high mortality rate. The Bahamas also had a high acceleration at 1.5. In total, 11 countries had a positive acceleration during the week of October 6, 2020, whereas only 6 countries had a positive acceleration for the week of October 13, 2020. The TAC displayed an overall positive trend, with a speed of 10.40, acceleration of 0.27, and jerk of –0.31, all of which decreased in the subsequent week to 9.04, –0.81, and –0.03, respectively.

**Conclusions:**

Metrics such as new cases, cumulative cases, deaths, and 7-day moving averages provide a static view of the pandemic but fail to identify where and the speed at which SARS-CoV-2 infects new individuals, the rate of acceleration or deceleration of the pandemic, and weekly comparison of the rate of acceleration of the pandemic indicate impending explosive growth or control of the pandemic. Enhanced surveillance will inform policymakers and leaders in the LAC about COVID-19 outbreaks.

## Introduction

### Background

**Figure 1:**
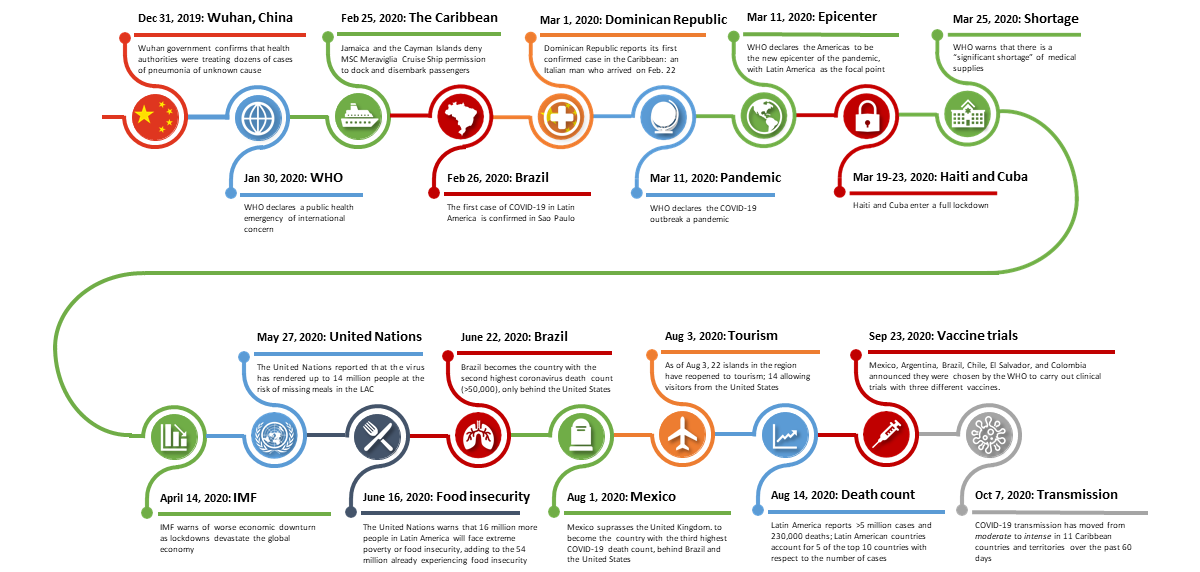
Timeline of the COVID-19 pandemic in Latin America and the Caribbean. IMF: International Monetary Fund, LAC: Latin America and the Caribbean, MSC: Mediterranean Shipping Company, WHO: World Health Organization.

The first confirmed case of COVID-19 in Latin America and the Caribbean (LAC) was an Italian national who entered the Dominican Republic on February 22, 2020, and tested positive for COVID-19 on March 1, 2020. By late May 2020, the World Health Organization declared the Americas as the new epicenter of the pandemic, with Latin America presenting particular concern [1] because by October 19, 2020, the World Health Organization confirmed 39,944,882 COVID-19 infections and 1,111,998 COVID-19–related deaths globally, of which 10,463,251 cases and 379,942 deaths were reported in the LAC [2]. Figure 1 shows the timeline of COVID-19 in the LAC.

Based on similar economies, geographies, and developments in the region, the World Bank defines the LAC to consist of Argentina, Belize, Bolivia, Brazil, Colombia, Chile, Costa Rica, Ecuador, El Salvador, Guatemala, Guyana, Honduras, Mexico, Nicaragua, Panama, Paraguay, Peru, Suriname, Uruguay, and Venezuela, Antigua and Barbuda, Aruba, the Bahamas, Barbados, Bermuda, the British Virgin Islands, Cayman Islands, Cuba, Curaçao, Dominica, Dominican Republic, Grenada, Haiti, Jamaica, Puerto Rico (the United States), Saint Kitts and Nevis, Saint Lucia, Saint Vincent and the Grenadines, Sint Maarten (the Netherlands), Trinidad and Tobago, Turks and Caicos Islands, and the US Virgin Islands [3]. As of this writing, the LAC represents many populations including the descendants of indigenous groups, European colonialists, enslaved Africans, and immigrants from East Asia and the Middle East, resulting in a shared history that still impacts social relations, economic structure, and political stability in the LAC [4]. Vulnerabilities within this shared history have become apparent during the COVID-19 pandemic, as manifested by climate change, poverty, food insecurity, political unrest, poor health care infrastructure, and economic instability, thus providing an important context in which transmission and surveillance of the SARS-CoV-2 must be understood [5].

### Economics

SARS-CoV-2 transmission has significantly affected the economies of several LAC countries. Approximately 700,000 jobs in Caribbean countries were lost between February and April, 2020, resulting in a projected 7.9% reduction in the gross domestic product (GDP), the largest decline recorded since 1930 [6]. Much of this loss stems from heavy dependence on tourism, which contributes to >25% of the average GDP and is the source of employment for >55% of the population in some Caribbean islands [7]. Containment measures, such as lockdowns, have devastated tourism-related businesses and depressed tax revenues. Latin America was already experiencing its lowest economic growth since the 1950s as the region is poised to face a 5.3% reduction in GDP [8]. The economic impact of the pandemic is amplified as nationwide lockdowns led to reductions in economic activity, global demand for regional exports, and commodity prices [9]. Nationwide lockdowns, quarantine, and curfew policies pose a particular issue for the 140 million informal workers in Latin America who are currently presented with limited employment opportunities [10]. Migrant remittances, another source of income for the most impoverished regions, are projected to decline by 20% [11]. Some aid comes from the United States or Europe, further compromising Latin America’s ability to combat the pandemic and its economic consequences [12]. Corruption and weak public health compliance has burdened health care systems with higher rates of disease transmission [13].

### Public Health Policies

Every Caribbean country with reported data adopted measures to limit the transmission of SARS-CoV-2. During the early stages of the pandemic, all countries implemented containment policies targeting the spread of COVID-19 through travel, tourism, and gatherings [14-16].

In June and July, many Caribbean countries reopened their borders to air travel subject to the following restrictions: (1) visitors are required to submit negative results on COVID-19 tests prior to arrival or within 7 days and (2) visitors from countries with high or increasing infection rates, including the United States, are prohibited entry [17]. However, these policies have not been able to prevent local transmission of SARS-CoV-2. Reports indicate that current transmission patterns are primarily driven by community spread with disease transmission due to tourism having less of an impact relative to the initial stages of the pandemic [18]. As of October 2020, these policies remain in flux and are likely to be revised as infection rates increase and decrease in the United States, Canada, European countries, and other countries that have historically accounted for large numbers of tourists to the Caribbean. 

Latin America represents a mosaic of failures and success stories regarding public health policies, displaying the varying effects of governmental unity and disunity. As an example of political disunity, as of December 11, 2020, Brazil has reported 6,728,452 cases and Mexico has reported 1,205,229 cases [2], both countries are affected by inadequate responses to the pandemic owing to delayed onset of public health guidelines, presidential apathy, premature reopening, and population density [19-21]. Mexican President Andrés Manuel López Obrador downplayed the severity of the virus and advocated for citizens to combat the disease at home, which led to death due to COVID-19 at home among many people in Mexico without confirmatory testing. This has underscored the true death toll even as Mexico is currently faced with an increasingly intense COVID-19 outbreak with 86,059 deaths. In a similar move, despite testing positive for COVID-19, Brazilian President Jair Bolsonaro consistently undermined quarantine, social distancing, and other public health measures by encouraging mass gatherings, dismissing the danger of the virus, promoting unproven remedies, and calling on citizens to go back to work in defiance of advice from the Brazilian Health ministry [22-24]. Owing to this intragovernmental conflict, the disease spread within these 2 countries, disproportionately affecting rural and indigenous populations [25,26]. For example, much of Brazil relies on the Amazon river to transport food, medicine, and emergency aid to 30 million people [27]. The indigenous people of Brazil have a 6-fold higher risk of COVID-19 compared to European, African, Asian, and other ethnic Brazilians not only owing to their vulnerability to infection given their paucity of exposure to outsiders [26], but also because government health workers are transmitting the virus because they do not have proper protective equipment or adequate access to tests, nor do they follow preventive measures recommended by public health authorities [28].

In contrast with Brazil and Mexico, Uruguay is a true success story in the region, as it was able to reopen public schools in June 2020, and is the only Latin American country whose locals are allowed to enter the European Union [29]. Uruguay implemented lockdowns in March as the government and public health experts presented a united front with consistent advice for the population [30]. With this political unity in combination with its robust national health system, Uruguay has been able to avoid the pitfalls that other Latin American countries have faced.

## Methods

The Foundation for Innovative New Diagnostics, Our World in Data, and The COVID Tracking Project [31-33] compile data from multiple sources across individual websites, statistical reports, and press releases; data for the most recent 7 weeks were accessed from the GitHub repository [34-36] using application programming interface. This yielded a panel of 32 countries in the LAC with 45 days in each panel (n=1440). An empirical difference equation was used, in which the number of positive cases in each country on each day is a function of the prior number of cases, the level of testing, and weekly shift variables that determine whether the contagion accelerated, decelerated, or remained unchanged compared to previous weeks. This resulted in a dynamic panel model that was estimated using the generalized method of moments approach by implementing the Arellano–Bond estimator in R [37-39]. Arellano–Bond estimation of difference equations has several statistical advantages: (1) it allows for statistical examination of the model’s predictive ability and the validity of the model specification, (2) it corrects for autocorrelation and heteroscedasticity, (3) it has favorable properties for data with a small number of time periods and large number of states, and (4) it corrects for omitted variables and provides a statistical test of correction validity [40,41]. With these advantages, the method is applicable to ascertaining and statistically validating changes in the evolution of the pandemic within a period of ≤1 week, such as changes in the reproduction rate.

## Results

### Country-Wise Regression Analysis

In accordance with the World Bank regional division, we grouped 32 countries into the broader LAC region. The results of the associative regression are reflected in Table 1 and are the basis of the weekly surveillance metrics.

As seen in Table 1, the Wald statistic for regression is significant (*χ*^2^_11_=932; *P*<.001) and the Sargan statistic for validity is nonsignificant (*χ*^2^_481_=32; *P*=.99), failing to reject the validity of overidentifying restrictions.

As shown in Table 1, the 1-day lag coefficient and the weekend effect are both nonsignificant. The 7-day lag coefficient is positive and significant (0.94; *P*<.001), suggesting a marked impact of infections from 1 week prior on infections at the time of data collection. The shift parameter for the week of October 6 is negative and significant (–1.36; *P*=.02), suggesting a rate change in disease transmission. The cumulative tests coefficient is slightly positive and significant (<0.001; *P*=.04).

**Table 1:** Arellano–Bond dynamic panel data modeling of the number of daily infections reported by Latin American and Caribbean countries from September 30 to October 13, 2020.

Variable	Coefficient (*P* value)
1-day lag coefficient	–0.16 (.08)
7-day lag coefficient	–0.94 (<.001)
Cumulative tests	<0.001 (.04)
Shift parameter for the week of October 6, 2020	–1.36 (.02)
Shift parameter for the week of October 13, 2020	–2.68 (.10)
Weekend effect	–0.08 (.56)
Wald statistic for regression (*χ*^2^ [df=11])	932 (<.001)
Sargan statistic for validity (*χ*^2^ [df=481])	32 (.99)

### Interpretation: Regression Analysis for the LAC

The lagging indicators and shift parameters suggest a recent change in disease transmission. As anticipated, the cumulative test effect is predictive of daily infections. No weekend effect was observed.

### Surveillance Results

Tables 2-7 include static and dynamic surveillance metrics for the weeks of October 6, 2020, and October 13, 2020. Static metrics include the number of new COVID-19 cases, cumulative COVID-19 cases, the 7-day moving average of new cases, the rate of infection, number of new deaths, cumulative deaths, the 7-day moving average of the number of deaths, and death rates (Tables 2 and 3). Novel dynamic metrics provide an overview of the impact of past cases on current cases and the potential trajectory of cases in the future. Novel dynamic metrics include (1) speed or the weekly average of new daily cases per 100,000 individuals, (2) acceleration or the day-to day-change in speed, (3) jerk or the week-over-week change in acceleration, and (4) the 7-day persistence effect or the number of new cases per 100,000 individuals reported thus far that are associated with new cases reported 7 days ago (Tables 4 and 5). The persistence effect is the only surveillance metric that controls for incomplete case ascertainment and data contamination. It also is consistent with superspreader events or an underlying condition that persists from the last week to the current week.

Static surveillance metrics are presented in Table 2 for the week of September 30 to October 6, 2020, and in Table 3 for the week of October 7 to 13, 2020. New cases in the region totaled to 79,053 on October 6, 2020, and to 42,837 on October 13, 2020. The 7-day moving average of new cases for the week of October 6, 2020, was 56,106 and that for the week of October 13, 2020, was 47,276. The total infection rate decreased from 12.42 per 100,000 population to 6.73 per 100,000 population, accompanied by a reduction in the mortality rate from 0.33 per 100,000 population to 0.24 per 100,000 population.

Within the region, on September 30, 2020, Brazil reported the largest number of new cases at 41,906, followed by Argentina at 14,740, Colombia at 7650, and Mexico at 4828 (Table 2). On October 7, 2020, Argentina reported the largest number of new cases at 13,305, followed by Brazil at 10,220, Colombia at 5014, and Mexico at 4295 (Table 3). For both weeks, Brazil had the highest 7-day moving average, followed by Argentina.

As shown in Table 2, the countries with the highest infection rates for the week of October 6, 2020, include Argentina at 32.8, the Bahamas at 27.5, Costa Rica at 20.1 and Brazil at 19.9. Infection rates, a measure of cases per 100,000 individuals in the population, generally decreased during the following week. For the week of October 6, 2020, death rates were the highest in the Bahamas at 1.03, Belize at 1.02, and Argentina at 0.8. Brazil and Costa Rica, while having some of the highest infection rates in the region, had markedly lower death rates of 0.39 and 0.34, respectively (all measured as cases per 100,000 population). With the exception of Argentina, where a slight increase was observed, death rates generally decreased during the following week.

During the week of October 6, 2020, (Table 4), the countries with the highest speed or average of new daily cases per 100,000 individuals in the population included Argentina at 27.9, the Bahamas at 24.1, and Costa Rica at 21.3, largely consistent with the infection rates during that week. All 3 countries had positive acceleration or change in speed during the week of October 6, 2020. These 3 countries also had the top speeds in the region in the following week, although the Bahamas had a reduction in speed to 22.2, while Costa Rica and Argentina had an increase in speed to 22.9 and 29.4, respectively. Mexico, while having a high number of cases in the region in accordance with its population, had a reduction in speed from 6.3 during the week of October 6, 2020, to 3.4 during the following week.

The utility of the speed metric is further enhanced by considering acceleration and jerk, which provide insight into potential infection trajectory. For the week of October 6, 2020, Belize had the highest acceleration and jerk in the region, at 1.7 and 1.8, respectively, which is particularly concerning considering its high mortality rate. The Bahamas also had a high acceleration of 1.5. In total, 11 countries had a positive acceleration during the week of October 6, 2020, whereas only 6 countries had a positive acceleration for the week of October 13, 2020 (Table 5). The LAC displayed an overall positive trend, with a speed of 10.40, acceleration of 0.27, and jerk of –0.31, all decreasing the subsequent week to 9.04, –0.81, and –0.03, respectively.

The 7-day persistence metric identifies the impact of the 7-day lag of speed on the current value of speed. New cases from 7 days prior have an echo effect, which impacts the current number of cases. Table 6 shows the countries with the highest 7-day persistence, which refers to the number of new cases reported per 100,000 population as of this writing, which is a result of new cases reported 7 days prior. Argentina maintained the highest 7-day persistence during both weeks, followed by Costa Rica, the Bahamas, and Panama.

Complete surveillance data for the LAC are provided in Multimedia Appendices 1-4.

**Table 2:** Static surveillance metrics for the week of September 30 to October 6, 2020.

Country	New weekly COVID-19 cases	Cumulative COVID-19 cases	7-Day moving average of new cases	Infection rate per 100,000 individuals	New weekly deaths	Cumulative deaths	7-Day moving average of deaths	Death rate per 100,000 individuals
Antigua and Barbuda	N/A^a^	107	0.86	N/A	N/A	3	N/A	N/A
Argentina	14,740	824,468	12,551.29	32.8	359	21,827	758.3	0.80
The Bahamas	107	4559	93.71	27.5	4	100	1.3	1.03
Barbados	N/A	200	1.43	N/A	N/A	7	N/A	N/A
Belize	47	2243	50.29	12.0	4	34	1.4	1.02
Bolivia	361	137,468	403.86	3.1	27	8156	32.1	0.23
Brazil	41,906	4,969,141	27,374.14	19.9	819	147,494	653.3	0.39
Chile	1,560	473,306	1715.14	8.2	33	13,070	49.3	0.17
Colombia	7650	869,808	6538.00	15.2	173	27,017	169.9	0.34
Costa Rica	1013	82,142	1076.86	20.1	17	1004	17.7	0.34
Cuba	38	5883	50.29	0.3	N/A	123	0.1	N/A
Dominica	N/A	31	0.14	N/A	N/A	N/A	N/A	N/A
Dominican Republic	317	115,371	495.86	3.0	5	2149	6.9	0.05
Ecuador	717	142,056	901.00	4.1	21	11,702	55.7	0.12
El Salvador	95	29,634	93.29	1.5	4	869	4.3	0.06
Grenada	N/A	24	N/A	N/A	N/A	N/A	N/A	N/A
Guatemala	688	94,870	557.43	4.1	8	3310	10.3	0.05
Guyana	N/A	3188	48.86	N/A	2	92	2.0	0.26
Haiti	11	8838	14.00	0.1	N/A	229	0.3	N/A
Honduras	642	80,662	652.00	6.6	14	2447	17.7	0.14
Jamaica	97	7109	100.14	3.3	3	123	3.1	0.10
Mexico	4828	794,608	8063.57	3.8	471	82,348	740.7	0.37
Panama	683	116,602	678.43	16.1	10	2440	10.9	0.24
Paraguay	932	45,647	792.29	13.2	19	966	17.9	0.27
Peru	1830	829,999	3040.71	5.6	92	32,834	72.9	0.28
St Kitts and Nevis	N/A	19	N/A	N/A	N/A	N/A	N/A	N/A
St Lucia	N/A	27	N/A	N/A	N/A	N/A	N/A	N/A
St Vincent and the Grenadines	N/A	64	N/A	N/A	N/A	N/A	N/A	N/A
Suriname	11	4965	14.57	1.9	N/A	106	0.3	N/A
Trinidad and Tobago	79	4846	54.71	5.7	1	83	1.3	0.07
Uruguay	22	2177	20.57	0.6	1	49	0.1	0.03
Venezuela	679	79,796	776.14	2.4	7	665	6.3	0.02

^a^N/A: not applicable.

**Table 3:** Static surveillance metrics for the week of October 7 to 13, 2020.

Country	New weekly COVID-19 cases	Cumulative COVID-19 cases	7-Day moving average of new cases	Infection rate per 100,000 individuals	New weekly deaths	Cumulative deaths	7-Day moving average of deaths	Death rate per 100,000 individuals
Antigua and Barbuda	N/A^a^	111	0.57	N/A	N/A	3	N/A	N/A
Argentina	13,305	917,035	13,223.86	29.6	386	24,572	392.1	0.86
The Bahamas	N/A	5163	86.29	N/A	N/A	108	1.1	N/A
Barbados	2	210	1.43	0.7	N/A	7	N/A	N/A
Belize	16	2585	48.86	4.1	2	39	0.7	0.51
Bolivia	227	138,922	207.71	2.0	25	8351	27.9	0.22
Brazil	10,220	5,113,628	20,641.00	4.8	309	150,998	500.6	0.15
Chile	1448	484,280	1567.71	7.6	20	13,396	46.6	0.11
Colombia	5014	924,098	7755.71	10.0	156	28,141	160.6	0.31
Costa Rica	1015	90,238	1156.57	20.1	16	1124	17.1	0.32
Cuba	17	6017	19.14	0.1	N/A	123	N/A	N/A
Dominica	N/A	32	0.14	N/A	N/A	N/A	N/A	N/A
Dominican Republic	165	119,008	519.57	1.5	4	2183	4.9	0.04
Ecuador	856	148,171	873.57	4.9	17	12,235	76.1	0.10
El Salvador	284	30,480	120.86	4.4	5	899	4.3	0.08
Grenada	N/A	25	0.14	N/A	N/A	N/A	N/A	N/A
Guatemala	554	98,380	501.43	3.3	23	3410	14.3	0.14
Guyana	44	3565	53.86	5.6	2	106	2.0	0.26
Haiti	5	8887	7.00	0.0	N/A	230	0.1	N/A
Honduras	439	84,852	598.57	4.5	7	2528	11.6	0.07
Jamaica	97	7910	114.43	3.3	N/A	146	3.3	N/A
Mexico	4295	825,340	4390.29	3.4	475	84,420	296.0	0.37
Panama	494	121,296	670.57	11.6	9	2511	10.1	0.21
Paraguay	853	51,197	792.86	12.1	12	1108	20.3	0.17
Peru	2803	853,974	3425.00	8.6	62	33,419	83.6	0.19
St Kitts and Nevis	N/A	19	N/A	N/A	N/A	N/A	N/A	N/A
St Lucia	N/A	29	0.29	N/A	N/A	N/A	N/A	N/A
St Vincent and the Grenadines	N/A	64	N/A	N/A	N/A	N/A	N/A	N/A
Suriname	14	5072	15.29	2.4	N/A	107	0.1	N/A
Trinidad and Tobago	11	5127	40.14	0.8	1	93	1.4	0.07
Uruguay	24	2337	22.86	0.7	N/A	51	0.3	N/A
Venezuela	635	84,391	656.43	2.2	6	710	6.4	0.02

^a^N/A: not applicable.

**Table 4:** Novel surveillance metrics for the week of September 30 to October 6, 2020.

Country	Speed: daily number of positive cases per 100,000 individuals (weekly average of new daily cases per 100,000 individuals)	Acceleration: day-to-day change in the number of positive cases per day (weekly average per 100,000 individuals)	Jerk: week-over-week change in acceleration (per 100,000 individuals)	7-Day persistence effect on speed (number of new cases per day per 100,000 individuals)
Antigua and Barbuda	0.9	0.0	0.0	0.1
Argentina	27.9	0.4	0.6	23.5
The Bahamas	24.1	1.5	0.0	13.7
Barbados	0.5	0.0	0.0	–0.5
Belize	12.9	1.7	1.8	7.8
Bolivia	3.5	–0.1	0.0	3.0
Brazil	13.0	0.7	0.7	10.7
Chile	9.0	–0.1	0.1	8.0
Colombia	13.0	0.5	0.0	11.2
Costa Rica	21.3	0.3	–0.5	19.5
Cuba	0.4	0.0	0.0	–0.2
Dominica	0.2	0.0	0.0	0.5
Dominican Republic	4.6	0.1	–0.3	2.5
Ecuador	5.2	–0.1	–0.1	5.4
El Salvador	1.4	–0.2	0.0	1.4
Grenada	0.0	0.0	0.0	–0.6
Guatemala	3.4	0.0	–0.1	2.8
Guyana	6.2	–1.1	–2.5	6.1
Haiti	0.1	0.0	0.0	–0.5
Honduras	6.7	0.1	1.0	4.4
Jamaica	3.4	-0.7	–0.5	4.4
Mexico	6.3	0.0	–2.7	2.7
Panama	16.0	0.4	0.7	13.2
Paraguay	11.2	0.5	1.0	9.0
Peru	9.4	–0.7	–1.3	15.1
St Kitts and Nevis	0.0	0.0	0.0	–0.6
St Lucia	0.0	0.0	0.0	–0.6
St Vincent and the Grenadines	0.0	0.0	0.0	–0.6
Suriname	2.5	–0.4	–0.7	1.7
Trinidad and Tobago	3.9	0.0	0.2	3.4
Uruguay	0.6	0.0	0.0	–0.2
Venezuela	2.7	–0.1	0.0	2.1

**Table 5:** Novel surveillance metrics for the week of October 7 to 13, 2020.

Country	Speed: daily number of positive cases per 100,000 individuals (weekly average of new daily cases per 100,000 individuals)	Acceleration: day-to-day change in the number of positive cases per day (weekly average per 100,000 individuals)	Jerk: week-over-week change in acceleration (per 100,000 individuals)	7-Day persistence effect on speed (number of new cases per day per 100,000 individuals)
Antigua and Barbuda	0.6	0.0	0.0	0.2
Argentina	29.4	–0.5	0.1	24.5
The Bahamas	22.2	–3.9	–5.5	21.0
Barbados	0.5	0.1	0.1	–0.1
Belize	12.5	–1.1	–0.1	11.0
Bolivia	1.8	–0.2	0.0	2.6
Brazil	9.8	–2.1	–1.9	11.0
Chile	8.3	–0.1	0.0	7.5
Colombia	15.4	–0.7	–0.9	11.1
Costa Rica	22.9	0.0	0.5	18.5
Cuba	0.2	0.0	0.0	–0.2
Dominica	0.2	0.0	0.0	–0.4
Dominican Republic	4.8	–0.2	0.1	3.5
Ecuador	5.0	0.1	0.1	4.1
El Salvador	1.9	0.4	0.6	0.7
Grenada	0.1	0.0	–0.1	–0.6
Guatemala	3.0	–0.1	0.0	2.4
Guyana	6.9	0.8	1.6	5.0
Haiti	0.1	0.0	0.0	–0.5
Honduras	6.1	–0.3	–0.2	5.4
Jamaica	3.9	0.0	0.1	2.5
Mexico	3.4	–0.1	2.7	5.1
Panama	15.8	–0.6	–0.2	13.7
Paraguay	11.3	–0.2	–0.4	9.5
Peru	10.5	0.4	2.5	7.8
St Kitts and Nevis	0.0	0.0	0.0	–0.6
St Lucia	0.2	0.0	0.0	–0.6
St Vincent and the Grenadines	0.0	0.0	0.0	–0.6
Suriname	2.	0.1	0.2	1.7
Trinidad and Tobago	2.9	–0.7	–1.4	2.9
Uruguay	0.7	0.0	0.0	–0.1
Venezuela	2.3	0.0	0.0	1.8

**Table 6:** Comparison of 7-day persistence.

7-Day persistence in the week of September 30, 2020		7-Day persistence in the week of October 6, 2020	
Argentina	23.5	Argentina	24.5
Costa Rica	19.5	Bahamas	21.0
Peru	15.1	Costa Rica	18.5
Bahamas	13.7	Panama	13.7
Panama	13.2	Colombia	11.1

**Table 7:** The most populous Latin American and Caribbean countries in 2020.

Country	Population as of 2020
Brazil	212,559,417
Mexico	128,932,753
Colombia	50,882,891
Argentina	45,195,774
Peru	32,971,854
Venezuela	28,435,940

## Discussion

### Principal Findings

The LAC comprises 32 countries and has an extremely varied response to the COVID-19 pandemic. Part of the variation is explained by the population density, being land-locked, or being on an island, while other variations are explained by national and subnational policies on COVID-19 control with various degrees of implementation and enforcement of lockdowns, quarantines, crowd control, hygiene, and social distancing. Caribbean countries are more likely to experience economic pressure to restart economies and lift travel restrictions to support tourism. Differences among LAC countries are further impacted by varying high rates of obesity and other chronic diseases, combined with a largely young population, thus affecting the severity of disease progression and the death rate.

Metrics such as new cases, cumulative cases, deaths, and 7-day moving averages provide a static view of the pandemic but fail to identify where and the speed at which SARS-CoV-2 infects new individuals, the rate of increase or reduction in speed between the current and subsequent weeks, and how the rate of acceleration increases or decreases, which would indicate impending explosive growth or control of the pandemic. It is important to monitor large caseloads; however, sole reliance on the number of new infections would limit the analysis of the pandemic to countries with large populations. While this information is necessary, it is not sufficient. Measures including the 7-day persistence provide retrospective context for current figures and help identify superspreader events for targeted intervention.

The entire LAC saw a reduction in speed, acceleration, and jerk for the week of October 13, 2020, compared to the week of October 6, 2020, accompanied by a reduction in new cases and the 7-day moving average. This is largely due to reductions in the number of infections in Brazil and Mexico, the 2 countries containing over 50% of the population in the region. However, Brazil continues to have the highest 7-day moving average in the region, >2-fold that of Argentina, the next highest in the region, for the week of October 6, 2020.

Colombia and Peru are among the top 5 most populous countries in the region and they showed minor increases in speed, as did Argentina. Argentina displayed the highest infection rate and speed in the region, which can be attributed to insufficient testing and extremely loose restrictions, allowing the pandemic to progress uninhibited. In contrast, Venezuela is progressing at one-tenth the speed of Argentina, with negative acceleration probably owing to an extremely intense military and government responses to the pandemic, led by Venezuelan security forces, which is not observed elsewhere in the LAC.

Brazil and Costa Rica have relatively high infection rates and speeds in the region, both ranking within the top 5 countries in the week of October 6, 2020. However, both countries have a death rate that is approximately one-third that of Argentina and the Bahamas, the 2 of which have the highest infection rates. In addition, Belize has a lower infection rate than the average infection rate of the LAC but has the second highest death rate in the region. These discrepancies between infection rates and death rates are driven by factors beyond age because Belize has a younger age structure.

In addition to Argentina, the Bahamas and Costa Rica had the top 3 infection rates in the region in the week of October 6, 2020. The static and novel surveillance metrics for these countries also had the top 3 speeds in the region and positive acceleration in the week of October 6, 2020, indicating future growth in COVID-19 caseloads. These 3 countries featured in the top 5 for 7-day persistence over both weeks, with the Bahamas and Argentina seeing an increase in the 7-day persistence week over week, suggesting that these countries are still experiencing echoes from previously high numbers of new cases. The Bahamas and Costa Rica have both loosened travel restrictions to protect their tourist-driven economy, which has resulted in higher COVID-19 caseloads.

Overall, there is reason to be cautiously optimistic as speed, acceleration, and jerk are displaying an overall downward trend. Countries with positive acceleration for the week of October 6, 2020, displayed reductions in acceleration to zero or negative values for the week of October 13, 2020. The number of countries with positive acceleration decreased from 11 for the week of October 6, 2020, to 6 for the week of October 13, 2020. While these reductions are notable, outbreaks and a reversion of trends are possible if the status quo changes.

### Comparison to Prior Studies

This study is part of the global SARS-CoV-2 surveillance project on policy, persistence, and transmission carried out at Northwestern Feinberg School of Medicine. This research program has developed novel surveillance metrics including speed, acceleration, jerk, and 7-day persistence and applied them to all global regions.

### Limitations

Our data are limited by variations in reporting practices across countries, both in terms of granularity and frequency. In addition, variation in testing practices and the health care infrastructure may impact the discrepancy in the number of reported cases and their true value. The data are reported at the national level, which prevents the analysis of subnational-level data.

### Conclusion

The LAC surveillance metrics suggest that the region as a whole is displaying a downward trend, largely owing to increased control over COVID-19 outbreaks in the most populous countries. However, certain countries, such as Brazil and Argentina, continue to struggle in controlling the pandemic. The overall progress is precarious, and without consistent measures to control the pandemic, is likely to give way to continued outbreaks.

## Appendix

Multimedia Appendix 1 LAC weekly statistics.

Multimedia Appendix 2 Weekly Latin America and Caribbean statistics by country.

Multimedia Appendix 3 Weekly LA 7 Day Persistence Map.

Multimedia Appendix 4 Weekly LA Acceleration Jerk Map.

## Abbreviations

GDP: gross domestic product
LAC: Latin America and the Caribbean

## References

[1] Boadle A WHO says the Americas are new COVID-19 epicenter as deaths surge in Latin America. Reuters.

[2] WHO Coronavirus Disease (COVID-19) Dashboard. World Health Organization.

[3] The World Bank in Latin America and the Caribbean. The World Bank.

[4] Stern SJ (2009). Paradigms of Conquest: History, Historiography, and Politics. Journal of Latin American Studies.

[5] Winn P (2006). Americas: The Changing Face of Latin America and the Caribbean.

[6] Meighoo K (2020). The Caribbean and Covid-19: not a health crisis, but a looming economic one. Round Table.

[7] (2020). Contraction of Economic Activity in the Region Intensifies Due to the Pandemic: It Will Fall -9.1% in 2020. Economic Commission for Latin America and the Caribbean.

[8] Economic Commission for Latin America and the Caribbean (2020). Report on the Economic Impact of Coronavirus Disease (COVID-19) on Latin America and the Caribbean. UN-iLibrary.

[9] (2020). COVID-19 in Latin America and the Caribbean: Regional socio-economic implications and policy priorities. OECD.

[10] Marquez PV, Aguilera SH, Calderon LB (2020). Have South and Central America become the new coronavirus (COVID-19) epicenter?. World Bank Blogs.

[11] (2020). World Bank Predicts Sharpest Decline of Remittances in Recent History. The World Bank.

[12] Blofield M, Hoffmann B, Llanos M (2020). Assessing the Political and Social Impact of the COVID-19 Crisis in Latin America. Social Science Open Access Repository.

[13] Miller MJ, Loaiza JR, Takyar A, Gilman RH (2020). COVID-19 in Latin America: Novel transmission dynamics for a global pandemic?. PLoS Negl Trop Dis.

[14] Murphy M, Jeyaseelan S, Howitt C, Greaves N, Harewood H, Quimby KR, Sobers N, Landis RC, Rocke KD, Hambleton IR (2020). COVID-19 containment in the Caribbean: the experience of Small Island Developing States. medRxiv. Preprint posted online June 2, 2020.

[15] Caribbean Countries reopening plans/initiatives post COVID-19. Caribbean Public Health Agency.

[16] Precautionary prevention measures implemented by Caribbean Countries (outside travel-related measures) because of COVID-19. Caribbean Public Health Agency.

[17] COVID-19 Information. U.S. Embassy in Barbados, the Eastern Caribbean, and the OECS.

[18] Charles J (2020). The Caribbean has reopened and COVID-19 is spreading — but one island is finding success. Miami Herald.

[19] Dyer O (2020). Covid-19 hot spots appear across Latin America. BMJ.

[20] (2020). Coronavirus: Mexico's death toll becomes world's third highest. BBC News.

[21] Andreoni M (2021). Coronavirus in Brazil: What You Need to Know. The New York Times.

[22] Phillips D (2020). Bolsonaro ignored by state governors amid anger at handling of Covid-19 crisis. The Guardian.

[23] Londoño E, Simões M (2020). Brazil President Embraces Unproven 'Cure' as Pandemic Surges. The New York Times.

[24] (2020). Coronavirus: Brazil's President Bolsonaro tests positive. BBC News.

[25] Masera O, Riojas-Rodríguez H, Pérez-Padilla R, Serrano-Medrano M, Schilman A, Ruiz-García V, de la Sierra L, Berrueta V Vulnerabilidad a COVID-19 en poblaciones rurales yperiurbanas por el uso doméstico de leña. Instituto Nacional De Salud Pública.

[26] Wallace S (2020). Disaster looms for indigenous Amazon tribes as COVID-19 cases multiply. National Geographic.

[27] Turkewitz J, Andreoni M (2020). The Amazon, Giver of Life, Unleashes the Pandemic. The New York Times.

[28] Manuela AE Brazil Health Care Workers May Have Spread Coronavirus to Indigenous People. The New York Times.

[29] Simon MF (2020). How tiny Uruguay, wedged between Brazil and Argentina, has avoided the worst of the coronavirus. The Washington Post.

[30] Gonzalez E, Harrison C, Hopkins K, Horwitz L, Nagovitch P, Sonneland HK, Zissis C (2021). The Coronavirus in Latin America. AS/COA.

[31] SARS-CoV-2 Test Tracker. Foundation for Innovative New Diagnostics.

[32] Ritchie H, Ortiz-Ospina E, Beltekian D, Mathieu E, Hasell J, Macdonald B, Giattino C, Appel C, Roser M, van Woerden E, Gavrilov D, Bergel M, Crawford J, Gerber M Coronavirus Pandemic (COVID-19): Statistics and Research. Our World in Data.

[33] Our data compilation is finished. Our research and analysis work continues through May. The COVID Tracking Project.

[34] COVID19Tracking/Covid-public-api. GitHub.

[35] FINDCov19TrackerData. GitHub.com.

[36] covid-19-data. GitHub.

[37] Hansen LP (1982). Large Sample Properties of Generalized Method of Moments Estimators. Econometrica.

[38] Oehmke J, Moss C, Singh L, Oehmke T, Post L (2020). Dynamic Panel Surveillance of COVID-19 Transmission in the United States to Inform Health Policy: Observational Statistical Study. J Med Internet Res.

[39] Oehmke J, Oehmke T, Singh L, Post L (2020). Dynamic Panel Estimate-Based Health Surveillance of SARS-CoV-2 Infection Rates to Inform Public Health Policy: Model Development and Validation. J Med Internet Res.

[40] Post LA, Argaw ST, Jones C, Moss CB, Resnick D, Singh LN, Murphy RL, Achenbach CJ, White J, Issa TZ, Boctor MJ, Oehmke JF (2020). A SARS-CoV-2 Surveillance System in Sub-Saharan Africa: Modeling Study for Persistence and Transmission to Inform Policy. J Med Internet Res.

[41] Post L, Marogi E, Moss CB, Murphy RL, Ison MG, Achenbach CJ, Resnick D, Singh L, White J, Boctor MJ, Welch SB, Oehmke JF (2021). SARS-CoV-2 Surveillance in the Middle East and North Africa: Longitudinal Trend Analysis. J Med Internet Res.

