# Supplementary figures and images for: Latin America and the Caribbean SARS-CoV-2 Surveillance: Longitudinal Trend Analysis
Source: JMIR Public Health Surveill. 2021 Apr 27;7(4):e25728. doi: 10.2196/25728 (PMC8083950; doi:10.2196/25728)

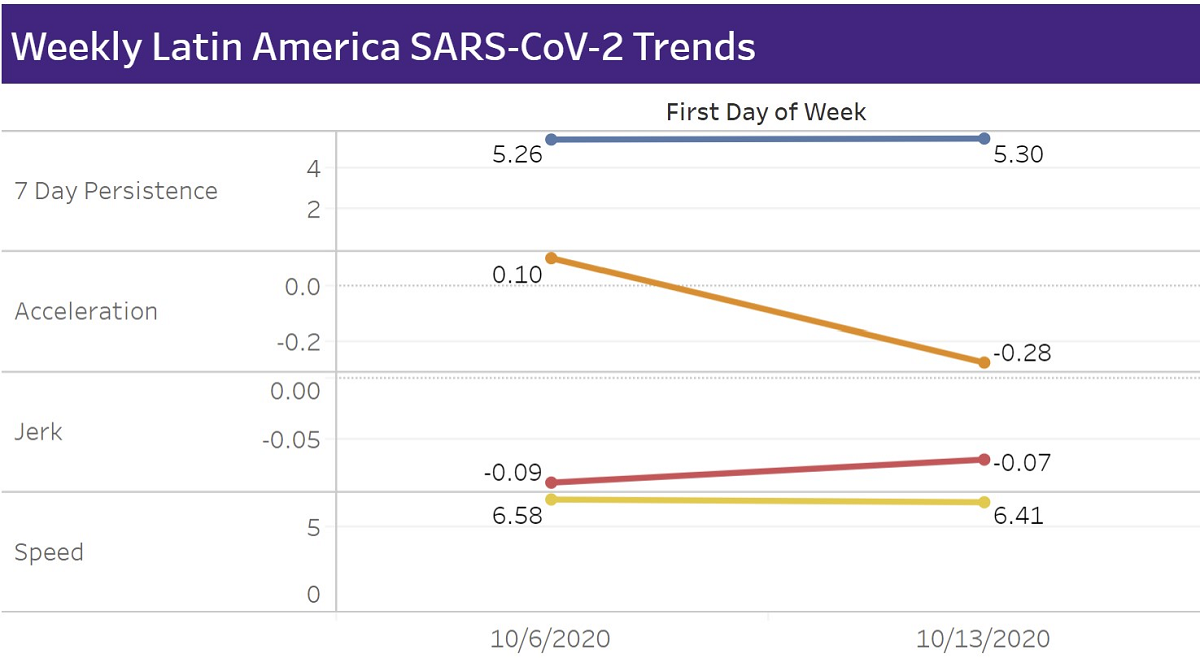

Supplement: Multimedia Appendix 1 [file publichealth_v7i4e25728_app1.png]

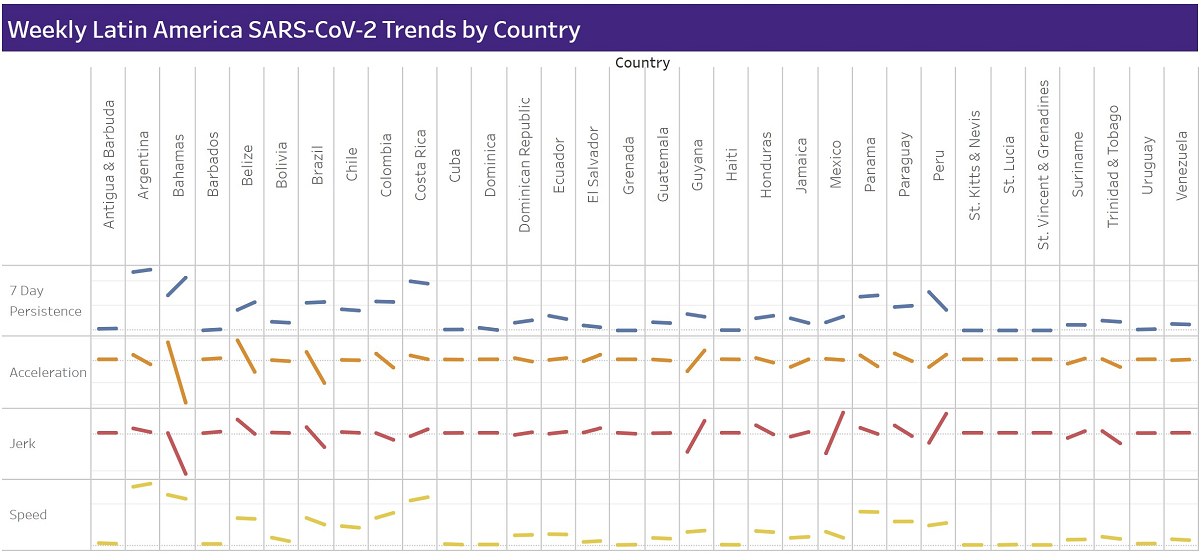

Supplement: Multimedia Appendix 2 [file publichealth_v7i4e25728_app2.png]

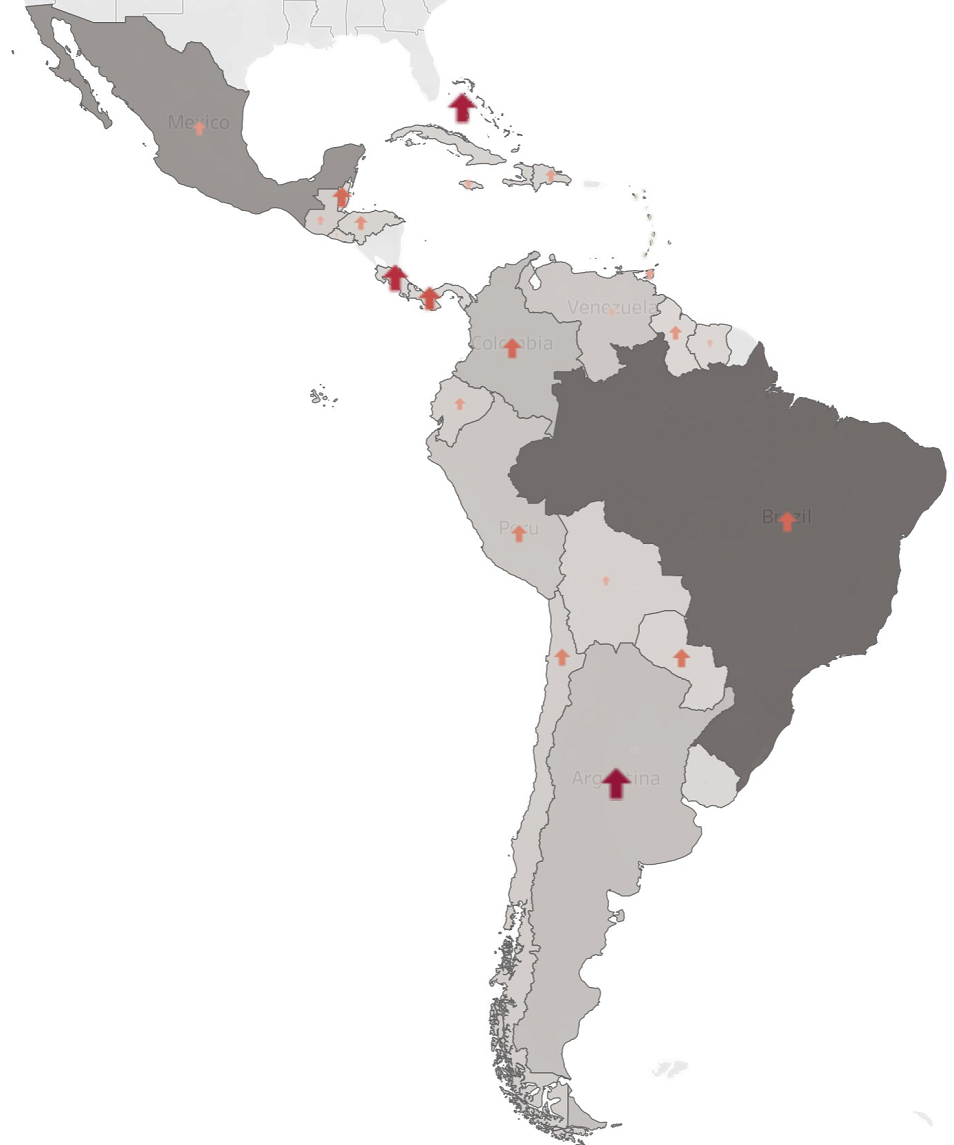

Supplement: Multimedia Appendix 3 [file publichealth_v7i4e25728_app3.png]

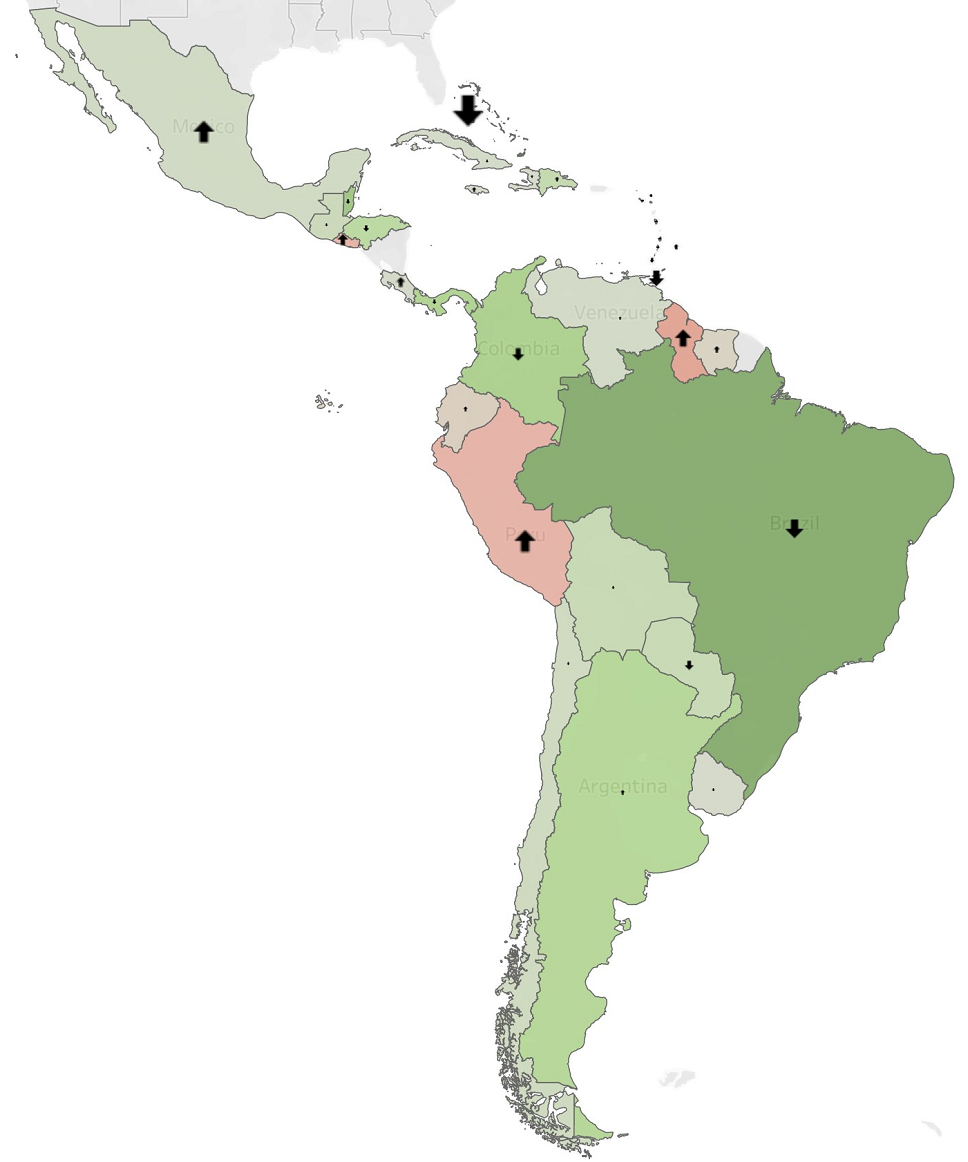

Supplement: Multimedia Appendix 4 [file publichealth_v7i4e25728_app4.png]
